# Optimization of multi-structural parameters in metamaterials based on the DGN co-simulation method

**DOI:** 10.1371/journal.pone.0328476

**Published:** 2025-07-23

**Authors:** Shangyang Jin, Fuxing Chen, Jie Bai, Bingfei Liu

**Affiliations:** 1 College of Safety Science and Engineering, Civil Aviation University of China, Tianjin, China; 2 China Southern Airlines Co Ltd, Guangdong, Guangzhou, China; 3 Institute of Science and Technology Innovation, Civil Aviation University of China, Tianjin, China; 4 College of Aeronautical Engineering, Civil Aviation University of China, Tianjin, China; Universiti Brunei Darussalam, BRUNEI DARUSSALAM

## Abstract

The convergence of algorithms is an unavoidable problem when using global optimization algorithms to optimize acoustic properties of metamaterials. The quality of optimization of local optimization algorithms is often limited by the initial data. Moreover, the influence of structural parameters on the performance is difficult to be reflected in the optimization process of traditional algorithms. Thus, a combination algorithm optimization strategy for metamaterials in terms of multiple structural parameters is proposed in this paper based on a co-simulation approach. This strategy combines the design of experiments (DOE), genetic algorithm (GA), and NLPQL algorithm, which is referred to as the DGN method. For the optimization problem of complex structures, firstly, the relationship between the structural parameters on acoustic performance can be obtained by fitting the relationship between design factors and the response function through DOE. Then the global algorithm is combined with the local algorithm to solve the problem of poor convergence of the global optimization algorithm while ensuring the optimization quality of the local optimization algorithm. Compared with the original structure, the optimized metamaterial structure has an optimization effect of 44.8% for the peak frequency position of sound insulation as well as an optimization effect of nearly 116.7% for the bandwidth of sound insulation. Compared with the optimization effect of single algorithm (NSGA-II), this method improves the optimization effect of acoustic isolation bandwidth by 36.8%. The optimized structure reflects better low-frequency sound insulation performance. Therefore, this optimization method provides a new idea for the design and performance regulation of metamaterials.

## Introduction

Researchers have shown considerable interest in developing lightweight low-frequency sound insulation materials to reduce the impact of low-frequency noise generated by ships or aviation equipment during operation. Traditional sound insulation materials are limited by the law of mass density [1–3], which requires increasing the surface density and weight of the structure to achieve low-frequency noise reduction. However, the emergence of acoustic metamaterials provides an innovative solution to this problem, offering new ideas and methods for reducing low-frequency noise [4–7].

Acoustic metamaterials have negative equivalent mass density and negative elastic modulus. Due to this double-negativity property the propagation of elastic waves in the medium is suppressed, thus achieving excellent low-frequency sound insulation performance. [1,8–11].

Due to their lightweight, ultra-thin structure, low-frequency properties, and variable, controllable design [12,13], membrane metamaterials have emerged as a primary area of interest in the study of low and medium-frequency noise reduction. As a result, they have become an important focus in the field of vibration and noise reduction.

In 2008, Yang, Mei [1] et al. introduced the concept of membrane acoustic metamaterials capable of violating the mass density law within the 100–1000 Hz range. Following that, they developed a dark acoustic membrane metamaterial that could absorb nearly 100% of acoustic waves in the low-frequency region [14].

In 2016, Chen et al. [15] developed a membrane-ring coupling structure that leverages both dipole and monopole resonances, resulting in a significantly broader attenuation bandwidth. In 2018, Wang et al. [16] designed a double-layer membrane acoustic metamaterial to integrate multiple impedance-coupled acoustic systems. Li et al. [17] created a honeycomb membrane acoustic metamaterial in 2020 that defies the law of mass and offers high sound transmission loss at a low weight. They also found that adjusting the structural parameters can alter the frequency of the peak sound insulation. In 2021, Huang et al. [18] designed a multi-resonator membrane metamaterial based on spider web topology, which offers a bionic approach to designing lightweight acoustic insulation structures with high functionality. Yao et al. [19] drew inspiration from the flexibility and versatility of cutting-edge origami design, a novel metamaterial with enhanced impact resistance capabilities was proposed. Inspired by ancient motifs, the auxetic model called auxetic structure (AS)-II was designed based on the combination of several promising mechanisms. And an auxetic meta-structure booster to advance piezoelectric energy harvesting was developed by Mohammad et al. [20]. Zha et al. [21] was inspired by Braess’s paradox, a novel mechanical model was proposed with reversible negative compressibility and filled the research gap in this aspect. Mohammad et al. [22] proposed a novel three-dimensional lightweight re-entrant meta-structure composed of a cross-shaped beam scatterer embedded in a host plate with holes based on the square lattice metamaterial and the drawback of narrow bandgaps of LRAMs has been solved. Salman et al. [23] presented the design of a locally resonant absorbing metamaterial in the form of cubes and rectangular cubes, formed from square and honeycomb shaped unit cells. It is possible to design stop bands with excellent performance in a certain targeted frequency range.

The above researches focus on the mechanism of low-frequency sound insulation properties and the design of metamaterial strategies to control the frequency range. And algorithms are used by scholars to realize the configuration design of the bandgap of phononic crystals, acoustic lenses [24,25], or acoustic cloaks [26]. And the Taylor approximation and approximate Newtonian optimization algorithm are devised by Chen et al. [27] to optimize the design of acoustic cloaks. Meanwhile, Lu et al. demonstrated designing the feasibility of mechanical metamaterials with negative Poisson’s ratio and high strength-to-weight ratio using the mixed-integer Bayesian optimization algorithm [28]. In 2023, the Kriging algorithm is utilized by Zhang et al. to create acoustic metamaterials that exhibited ultra-wide low-frequency bandgaps [29]. Peyman et al. [30] investigated quasiperiodic energy harvesting in a nonlinear vibration-based harvester, consisting of delayed nonlinear vibrations from magnetic levitation subjected to harmonic base acceleration, in which time-delay was introduced. And contributed to research with time-delays. To ameliorate the curse of dimensionality of SBO, Zhao et al. [31] proposed a supervised nonlinear dimensionality-reduction surrogate modelling method and significantly improved the modelling efficiency and accuracy. Mollajafari et al. [32] summarized that the integration of evolutionary algorithms and nature-inspired heuristic optimization drawing inspiration from biological processes have been instrumental in optimizing complex engineering problems.

By introducing algorithms into the structural design of metamaterials, the properties of metamaterials can be better regulated. However, it is difficult to reflect the influence of structural parameters on the optimization objectives in the optimization process of traditional algorithms. For the global optimization algorithm, despite the good optimization effect, its convergence is poor. Although the local optimization algorithm has good convergence, the quality of optimization is often limited by the initial data because it is easy to fall into local solutions.

Therefore, based on the co-simulation method, the Design of Experiments (DOE), Genetic Algorithm (GA), and NLPQL algorithm are combined in this paper. Based on the strategy of this combined algorithm, such an optimization method is referred to as the DGN method in this paper. In this method, the relationship between the influence of each structural parameter on the optimization objective can be analyzed, so that the structural parameters of metamaterials can be adjusted more conveniently. Furthermore, for the performance optimization of complex structures, the method can obtain a global better solution by genetic algorithm to ensure the optimization quality of the local optimization algorithm. Then the excellent local optimization ability of the local optimization algorithm is used to make the algorithm converge and improve the optimization effect. It is shown that based on the global optimization algorithm, the NLPQL algorithm achieves the iterative convergence of the algorithm with only about 30 cycles, which greatly improves the computational efficiency. The optimized metamaterial structure increases the acoustic bandwidth by 70 Hz and decreases the peak frequency of the acoustic isolation by 150 Hz compared with the original structure. Compared with the optimization result of the single algorithm, the acoustic isolation bandwidth of the optimized structure is increased by 35 Hz, and the frequency of the acoustic peak is decreased by 25 Hz. Therefore, the DGN method has great potential to be applied to the structural design and performance regulation of metamaterials.

## Optimization flow of DGN method based on co-simulation

**Fig 1:**
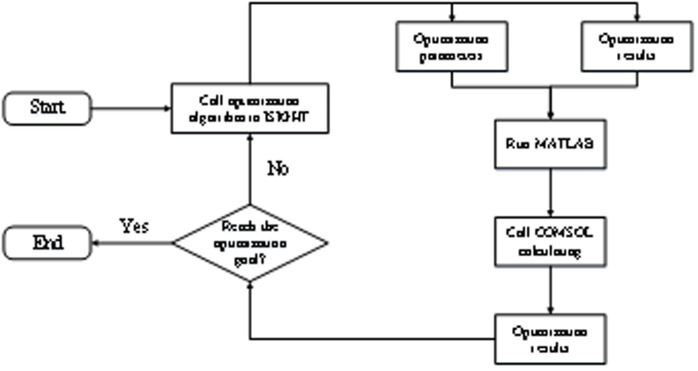
Optimization flow of the co-simulation method.

Based on the ISIGHT multidisciplinary optimization platform, this paper builds a joint simulation optimization process, calls the algorithm toolbox inside the ISIGHT software, and emembedded it into the operation of COMSOL to automatically optimize and calculate multiple structural parameters, achieving the regulation of the metamaterial band gap. The application of the joint simulation method to optimize the metamaterial structure not only avoids complex algorithm programming but also greatly reduces the workload. Moreover, based on the visualization platform and the feature of real-time data transmission optimization, the optimization process can be analyzed and evaluated in real time, greatly improving work efficiency. The optimization process based on the joint simulation method first conducts parametric modeling of acoustic metamaterials in COMSOL to determine the structural parameters that need to be optimized. And through programming, use MATLAB to read the structural parameters that need to be optimized and control the operation and solution of COMSOL, and output the calculation results of the model. Subsequently, through the relevant functional components of ISIGHT, the MATLAB program was integrated into the ISIGHT optimization platform, and the optimization parameters and calculation results of the model were imported and read. Finally, through the optimization module in ISIGHT, various optimization algorithms are called and the optimization objectives and algorithm parameters are set, thereby embedding the algorithms into the operation and solution of COMSOL to achieve the automatic optimization calculation of the structural parameters of metamaterials. The optimization process of the joint simulation method is shown in Fig 1. During the process of joint simulation optimization, MATLAB, as a “bridge”, combines COMSOL with ISIGHT to achieve the transmission between data. The optimization process setup in ISIGHT is shown in Fig 1. The main function of the Simcode component is to run the MATLAB program and read the optimization parameters and result files. The function of the Data-Matching component is to plot the corresponding curve based on the calculation results and determine the optimization function; The function of the Optimization component is to invoke the optimization algorithm and set the optimization objective.

The combined algorithm strategy mainly first obtains samples through the design of experiments method, utilizing the Latin hypercube algorithm to fit the relationship between the design factor and the objective function. Then the genetic algorithm and local optimization algorithm are combined to automatically optimize the structural parameters of acoustic metamaterials to improve the low-frequency acoustic isolation performance of metamaterials. Therefore, based on the combination of the design of experiments (DOE) method, GA, and NLPQL algorithm, the optimization method is referred to as the DGN optimization method.

**Fig 2:**
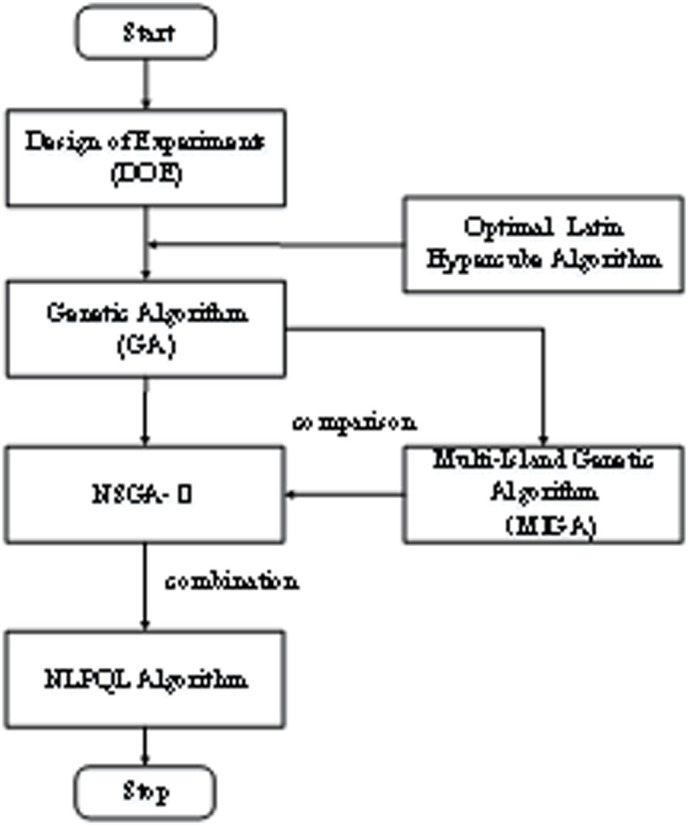
Optimization process of the DGN method.

The optimization flow of the DGN method is shown in Fig 2. In which the design of experiments (DOE) method is to conduct reasonable sampling within the range of design variables of the model through mathematical theories such as probability theory and statistics. Based on these sample points, a fitting analysis of the design spatial characteristics of the model is conducted to obtain the influence law of the design variables on the target characteristics. By using the experimental design method to analyze the model, not only can the response relationship of each design variable to the objective function and the influence among the design variables be obtained, but also the key variables affecting the objective function can be found through the response relationship and reasonable parameter adjustment can be carried out. For more complex metamaterial structures or multiple structural parameters, there is often mutual influence among the parameters. Adjusting the acoustic performance of metamaterials by parameter tuning may require a considerable amount of computing time. Therefore, by applying optimization algorithms, the multi-structural parameters of metamaterials can be automatically optimized and calculated to meet the design requirements, and the intelligent design of metamaterial structures can be achieved.

For the parameter adjustment problem of the complex structure, the global optimization algorithm and the local optimization algorithm are combined in this paper to realize the optimization calculation of the parameters. First, the GA is used to obtain the global better solution to ensure the optimization quality of the local optimization algorithm. Finally, the local optimization algorithm (NLPQL) helps the global optimization algorithm to converge and improve the optimization effect of the algorithm by its efficient local optimization ability and convergence. The combination of a genetic algorithm (GA) for global search and Nonlinear Programming by Quadratic Lagrangian (NLPQL) for local refinement leverages the strengths of both optimization techniques, making them highly complementary in solving complex, nonlinear optimization problems. GA is a population-based stochastic search method that explores a wide range of solutions across the search space, avoiding premature convergence to local optima. It means GA provides a set of good candidate solutions that are likely near the global optimum, even if not highly precise. NLPQL is a gradient-based local optimizer that refines solutions efficiently by leveraging quadratic approximations of the Lagrangian function. Once in the vicinity of a good solution, NLPQL can converge rapidly to a high-precision local optimum. Therefore, they complement each other by a global-to-local strategy: GA first performs a broad exploration to identify promising regions in the search space. And NLPQL then takes the best solutions from GA and fine-tunes them to achieve high accuracy. Together, they ensure both global robustness and local precision and reduce computational cost compared to running GA until full convergence. Furthermore they avoid the need for multiple random restarts in gradient-based methods. Therefore, the DGN method can greatly improve the optimization efficiency of the algorithm under the condition of ensuring the optimization quality.

## Application of DGN optimization methods to acoustic metamaterial

### Membrane acoustic metamaterial structure

**Fig 3:**
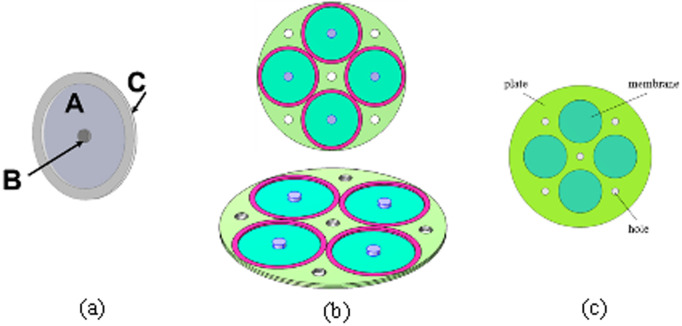
The membrane metamaterial structure.

To demonstrate the effectiveness of the DGN optimization method, a classical membrane acoustic metamaterial is chosen in this section. As shown in Fig 3a, this basic structure consists of three parts: A is the membrane, B is the center mass block, and C is the support ring. The membrane acoustic metamaterial is arranged in order as shown in Fig 3b, where the cyan part is the membrane (A), the blue part is the additional mass block (B), and the red part is the support ring (C). By making holes in the circular plate (white part), a through-hole membrane acoustic metamaterial is formed. The through-hole type membrane acoustic metamaterial excluding the additional mass block is shown in Fig 3c.

### Simulation and computation of the model

The original structure is shown in the Fig 3a. The simulation is carried out based on the parameters described in the literature [33–37]. The membrane employed for this experiment is composed of polyetherimide PEI and has a thickness of 0.0762 mm and an effective diameter of 24 mm. The material properties of the membrane include a modulus of 6.9 × 10^9^ Pa, a density of 1200 kg/m^3^, and a Poisson’s ratio of 0.36. Specifically, the model selected for the simulation had central mass blocks weighing 0.16g and 0.48g. The experiment of the TL for the structures was conducted by Naify et al. using the impedance tube (Brüel and Kjær model 4206). Its experiments were performed by placing two microphones upstream of the sample to measure the incident sound pressure level, while two microphones were located downstream. the transmission loss (TL) can be calculated as Equation (1) by measuring the displacement or sound pressure at the excitation and response points.


$$TL=20\log\frac{P_{i}}{P_{0}}$$ (1)

where $P_{0}$ and $P_{i}$ represent the amplitude of the transmitted and incident waves, respectively.

**Fig 4:**
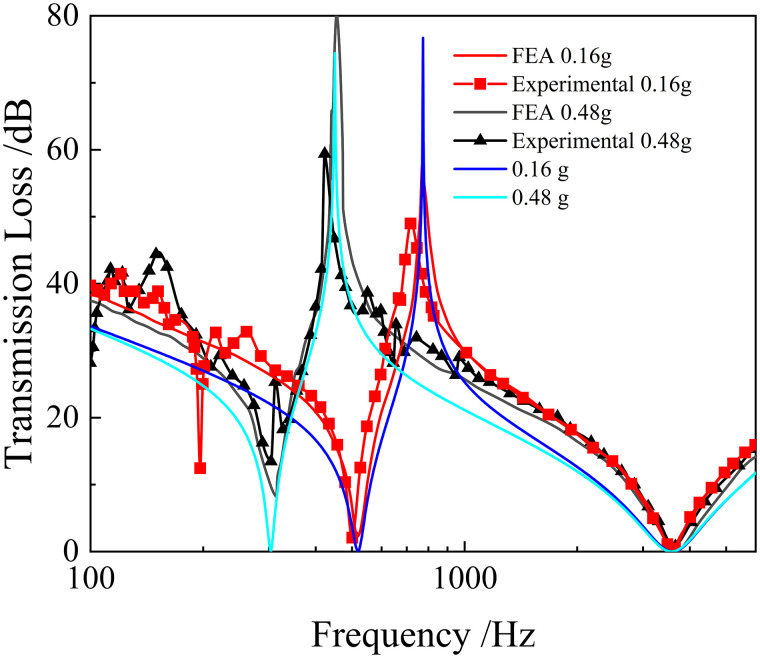
The TL of the metamaterial.

The sound insulation performance of the model was presented in Fig 4, with the red (FEA 0.16g and Experimental 0.16g) and black (FEA 0.48g and Experimental 0.48g) lines representing the results from the literature [34].

The blue (0.16g) and green (0.48g) lines are the simulation results by finite element analysis (FEA) using the data from the literature. The comparison between the simulation results in this study and those in the literature [34] (black and red lines) is shown in Fig 4. Furthermore, Table 1 presents the comparison of FEA results in the literature and the experimental results in the literature, with the FEA results shown in parentheses. Table 2 presents the comparison of FEA results (in parentheses) in this work and the experimental results in the literature.

**Table 1:** Comparison between FEA and experimental results [33].

Mass (g)	First resonance frequency (Hz)	TL peak frequency (Hz)	Second resonance frequency (Hz)
0.16	504 (530)	724 (790)	3632 (3750)
error	5.18%	9.11%	3.24%
0.48	300 (310)	428 (450)	3664 (3690)
error	3.33%	5.14%	0.71%

**Table 2:** Comparison between FEA (in this work) and experimental results [33].

Mass (g)	First resonance frequency (Hz)	TL peak frequency (Hz)	Second resonance frequency (Hz)
0.16	504 (518)	724 (774)	3632 (3596)
error	2.77%	6.91%	0.991%
0.48	300 (304)	428 (450)	3664 (3572)
error	1.33%	5.14%	2.51%

Table 2 shows that the simulation error in this paper is very small compared to the experimental results mentioned in the literature [34]. In addition, the simulation results in this paper are more accurate as compared to the simulation results in the literature [34]. As a result, the sound insulation performance curves are in good agreement with the experiment, further confirming the correctness of our simulation approach.

### _Insulation loss (TL) of the perforated structure_

The perforated structure is shown the Fig 3c, with a panel thickness of 2 mm and a radius of 59 mm, featuring four large holes with an 18 mm radius. Each of these large holes is equipped with a 1 mm-thick membrane. Additionally, there are five smaller ventilation holes with a radius of 3 mm. The material characteristics of the components are presented in Table 3.

**Table 3:** The material characteristics of the components.

Components	Young’s modulus	Poisson’s ratio	Density
Plate	205Gpa	0.3	7850 kg/ m^3^
Membrane	5.5Gpa	0.39	1432 kg/ m^3^

**Fig 5:**
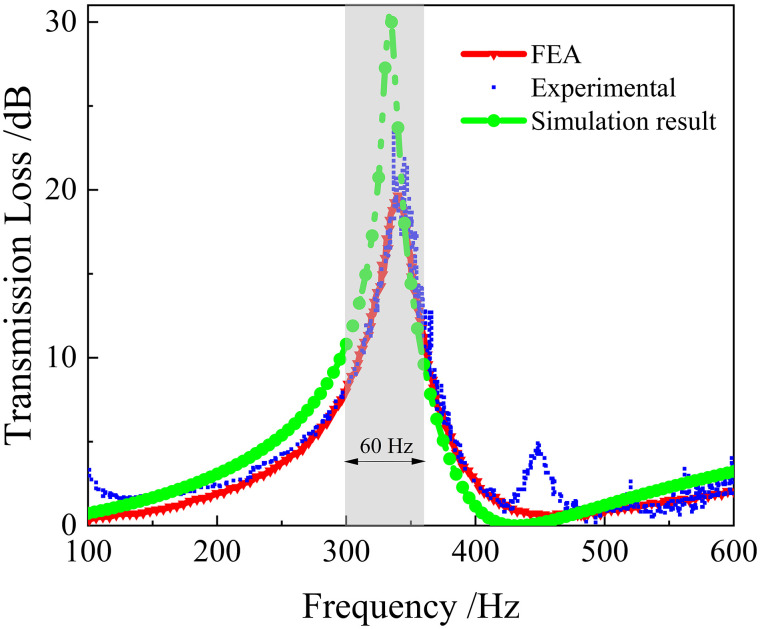
The TL of the perforated membrane metamaterial.

The Fig 5 displays the TL of the perforated membrane metamaterial, which exhibits a satisfactory concurrence between the simulation and experimental results. Moreover, the model’s replication results correspond well with those reported in literature [38]. The shaded portion in the figure represents the region where the sound insulation exceeds 10 dB.

This structure exhibits sound insulation of more than 10 dB within the 300–360 Hz, with a bandwidth of 60 Hz frequency range. The maximum insulation of 40.78 dB is attained at approximately 335 Hz.

**Fig 6:**
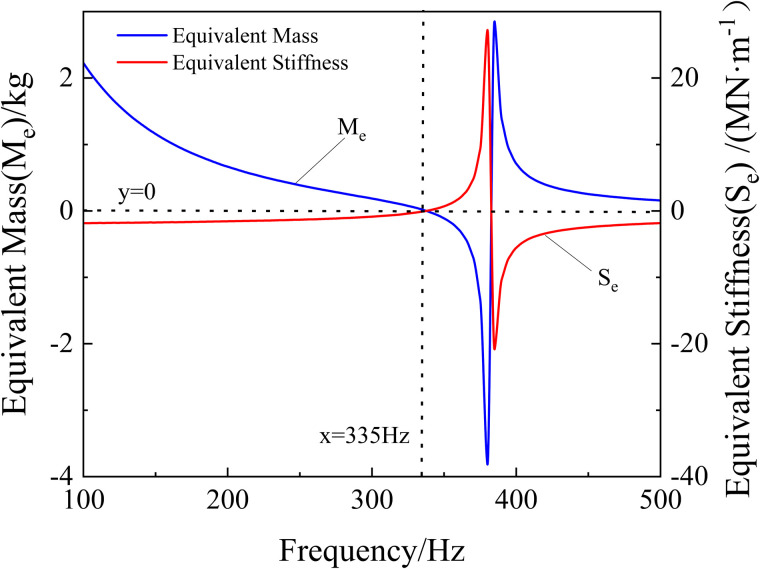
The equivalent mass and stiffness of the metamaterial.


The equivalent mass and equivalent stiffness of the membrane metamaterial are calculated by Equations (2) and (3). This metamaterial exhibits the negative equivalent parametric properties as shown in Fig 6. This is consistent with the sound-insulating metamaterials studied in the existing literature [1,8-11].

$$M_{e}=\frac{\int P_{i}-\int P_{0}}{a_{z}}$$ (2)

$$S_{e}=\frac{\int P_{i}-\int P_{0}}{d_{z}}$$ (3)

where the $a_{Z}$ and $d_{Z}$ are the average acceleration and average displacement of the metamaterial in the direction of incidence, respectively.

The results show that in the bands of 335–380 Hz, $M_{e}$ is negative. In the bands from 100 to 335 Hz and above 385 Hz, $S_{e}$ is negative. Therefore, the 335 Hz is the zero-value transition point of the $M_{e}$ and $S_{e}$ curves. Besides, there is an intersection point of the two curves at 380–385 Hz. And near this point, there is an extreme shift in both curves. The positive and negative $M_{e}$ are related to the direction of $a_{Z}$, while $S_{e}$ is related to $d_{Z}$. At the point of extreme shift, both $a_{Z}$ and $d_{Z}$ converge to 0, when the quasi-dynamic equilibrium point is reached. Therefore, near the equilibrium position, the excitation of metamaterials is difficult., and the sound insulation value reaches the maximum at this time. And this is consistent with the result that the insulation loss curve reaches its highest at around 335–360 Hz in Fig 6.

## _Optimization study based on co-simulation method_

### _Design of experiment method_

**Fig 7:**
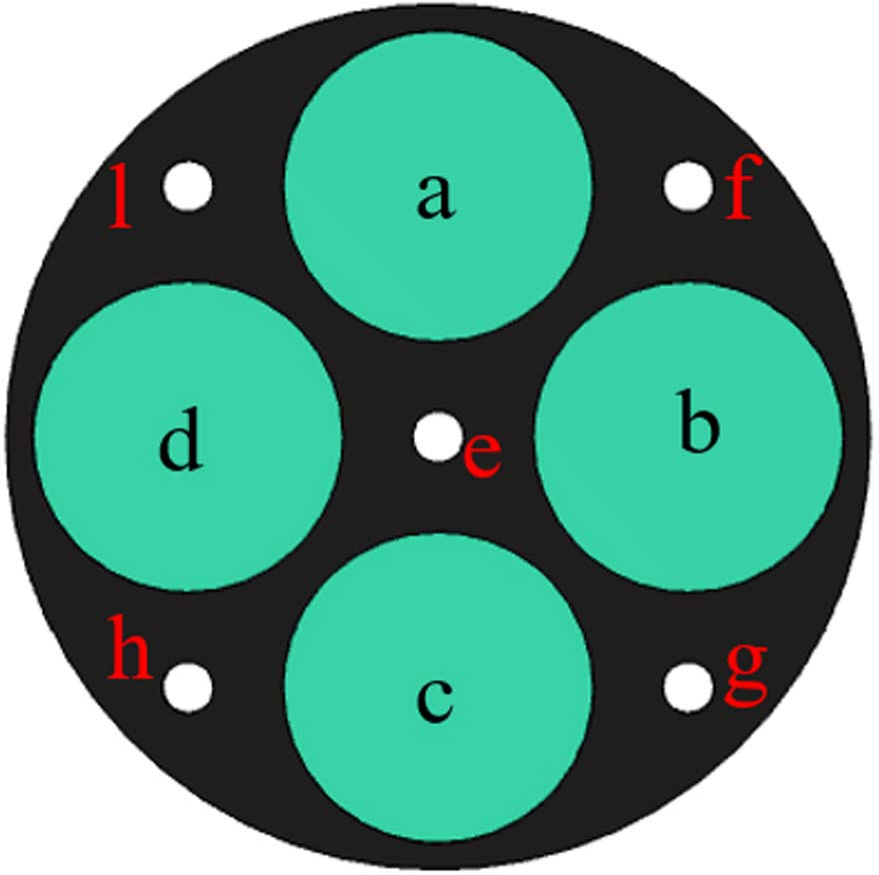
The nine structural parameters as design factors.

To further investigate the effects of multiple structural parameters on the acoustic insulation performance of metamaterials, the design-of-experiment (DOE) method is used in this section to derive the relationship between the parameters and the performance response function and to check the validity of the co-simulation method. As shown in Fig 7, the radii of nine circles in the circular plate are used as the design factors (a-l).

The optimal Latin hypercube sampling algorithm is selected in the experimental design. By the Latin hypercube algorithm (LHS), 180 sample points are taken in the design space and the selected sample points are simulated. LHS is a stratified sampling technique used to efficiently explore the parameter space of a computational model. It ensures that each parameter is divided into N equally probable intervals, and exactly one sample is placed in each layer. This provides better coverage than pure random sampling, especially in high dimensional spaces. However, standard LHS does not guarantee optimal uniformity in the multidimensional space. Which means some samples may still cluster undesirably when projected into higher dimensions. Optimal LHS (OLHS) is an enhanced version of LHS where an optimization criterion is applied to improve the sampling distribution. The goal is to minimize undesirable clustering and maximize space-filling properties. Standard LHS can sometimes introduce spurious correlations between variables, which OLHS mitigates. OLHS refines standard LHS by enforcing additional uniformity and space-filling criteria, making it superior for applications requiring high-fidelity sampling. This new sampling mechanism results in a more homogeneous and stable distribution of the sample space to ensure a more accurate matching of the design parameters and response functions.

The target response functions are set as follows:


$$\left\{ \begin{array}{c} 1.Maximize\;f(y)\\ 2.Minimize\;f(x)\\ 3.Maximize\;f(s) \end{array}\right.$$

where: 1. the f(y) is the TL peak; 2. the f(x) is the frequency of the TL peak; and 3. the f(s) is the area of the TL curve enclosed by the coordinate axis. When the overall performance of TL is improved, the area of the curve enclosed by the TL curve and the axes increases, so the area can be used to describe the overall sound insulation performance of the metamaterial in a certain frequency range (100–600 Hz).

The constraints for the parameters are set as follows:


$$\left\{ \begin{array}{c} 16\leq a,b,c,d,\leq 20\\ 1\leq e,f,g,h,l\leq 6 \end{array}\right.$$

The results of the analysis of each response function are as follows, where the values represent the magnitude of the effect of the structural parameters on the objective function.

For 1.Maximizef(y), only parameters a, c and parameter d have a positive effect (the larger the parameter, the larger the response function, which means that the peak acoustic isolation increases), the rest of the parameters have a negative effect on the response function.

For 2.Minimizef(x), increasing the parameters e, f, g, h, and l will cause the peak sound insulation to shift toward lower frequency, and the rest of the parameters will have the opposite effect.

For 3.Maximizef(s), all parameters have a negative effect on the target response function, which means that the increase of a single parameter decreases the acoustic isolation of the metamaterial. In this paper, the effect of change in the radius of a single inner and outer circular hole on its acoustic performance is investigated, as shown in Figs 11 and 12. Where the bars represent the frequency bandwidth of sound insulation of the metamaterial greater than 10 dB. It can be seen that the acoustic isolation bandwidth of the metamaterial decreases gradually with the increase of the hole diameter in the range of 100–600 Hz at low frequency, which indicates the decrease of its low-frequency acoustic isolation performance. This is also consistent with the results analyzed by the DOE method in Fig 10.

**Fig 8:**
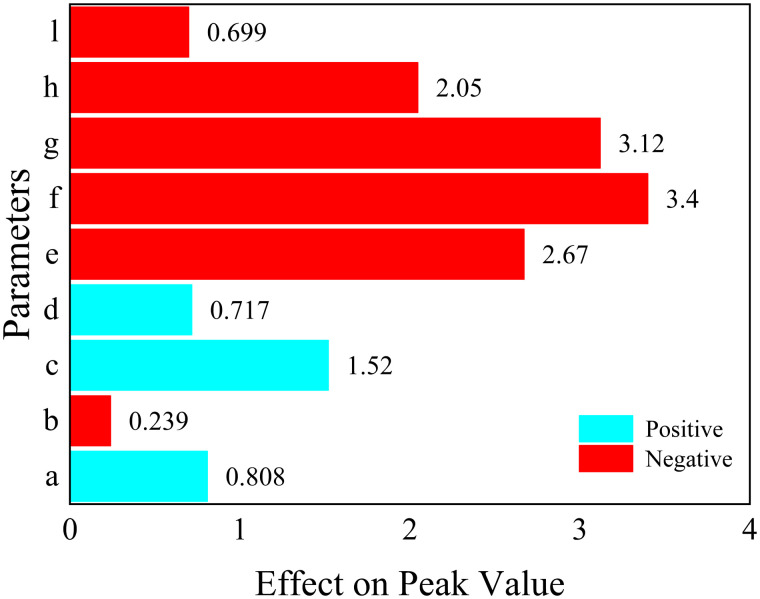
The effect of each parameter on the peak value.

**Fig 9:**
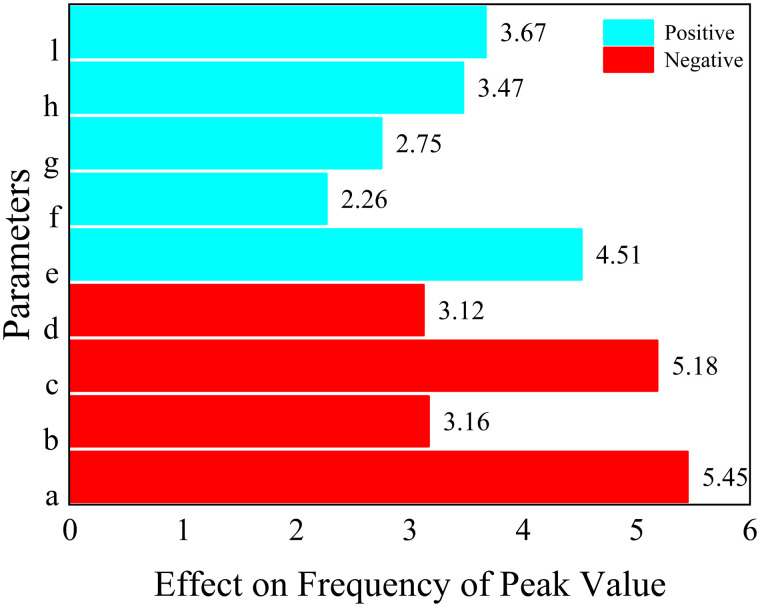
The effect of each parameter on the frequency of peak value.

**Fig 10:**
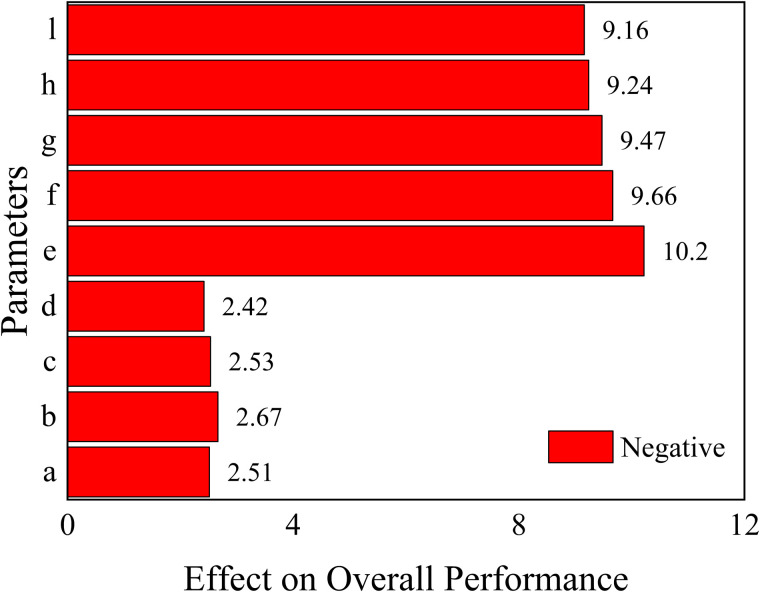
The effect of each parameter on the overall performance.

**Fig 11:**
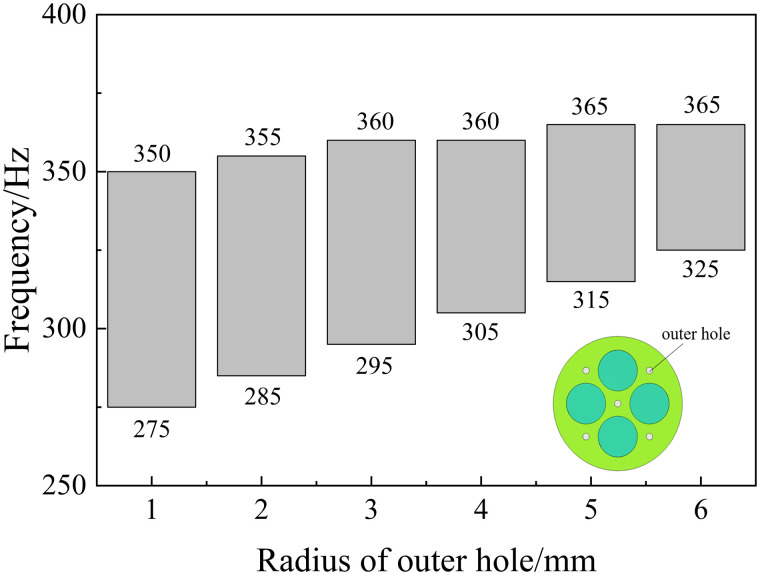
Effect of inner hole radius on sound insulation performance.

**Fig 12:**
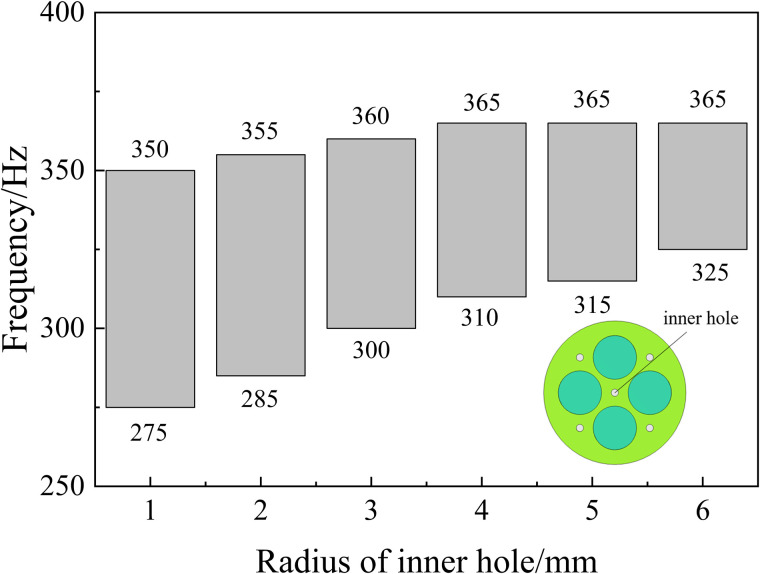
Effect of outer hole radius on sound insulation performance.

However, it is worth noting that Figs 8–10 only represent the influence factors of individual structural parameters on the target response function. The results in Figs 11 and 12 also analyze the influence of single structural parameter on the acoustic performance obtained by controlling variables.

**Fig 13:**
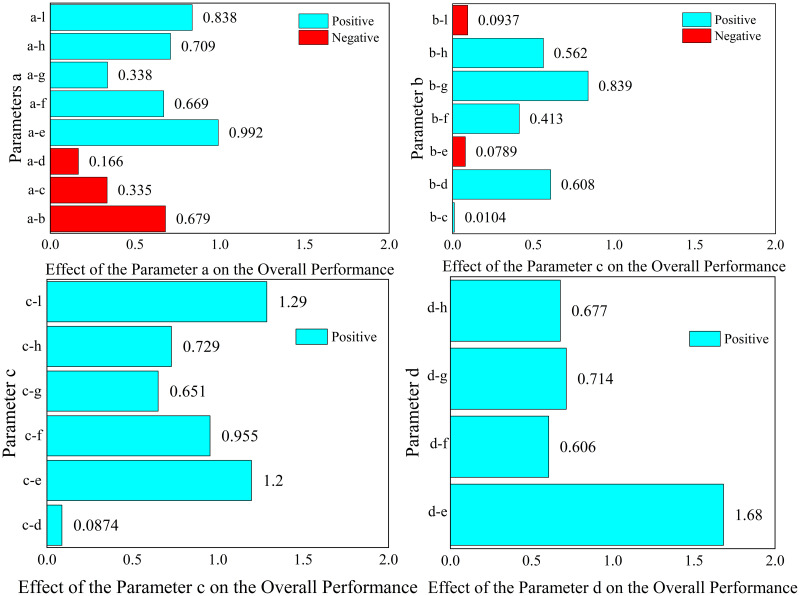
The combined effect between each parameter on the target 3.

As for the more complex metamaterial structures, there is also a mutual influence or even a mutual constraint relationship among the parameters. For the response objective function 3.Maximizef(s), the relationship between the parameters is obtained by the DOE method is shown in Fig 13.

**Fig 14:**
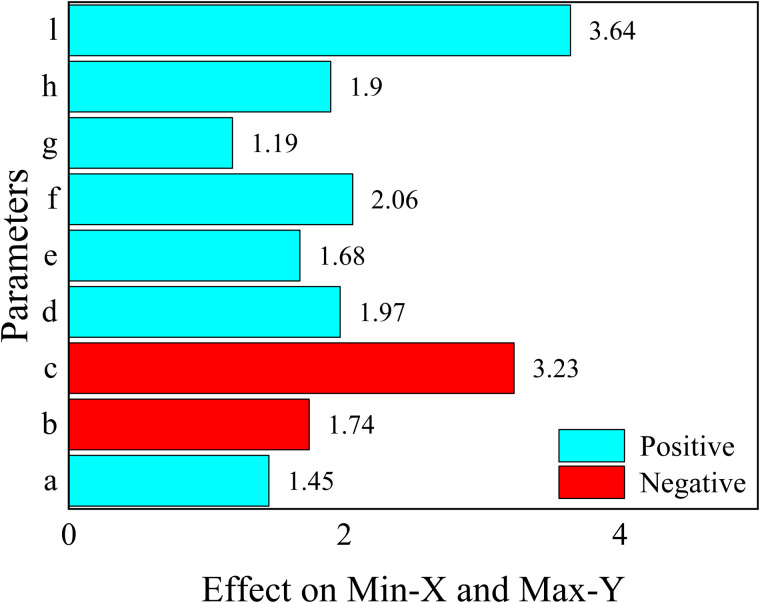
The effect of each parameter on the target functions.

**Fig 15:**
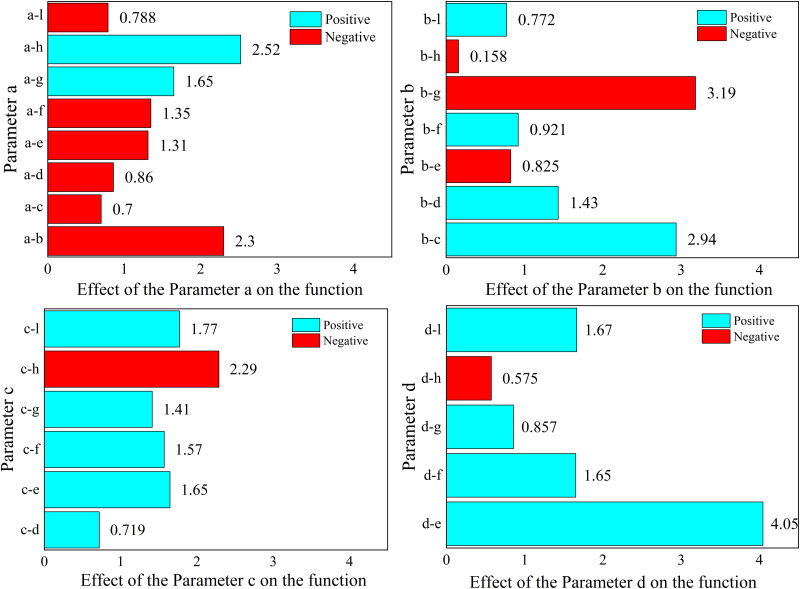
The combined effect between each parameter on the target functions.

If the response functions of several targets are considered at the same time, the 1.Maximizef(y) and the2.Minimizef(x) response functions can be combined at the same time. This means that the peak frequency of the sound insulation is reduced while ensuring that the peak sound insulation is maximized. The reduction of peak frequency is beneficial to human health and material safety. Considering of human hearing sensitivity, lower-frequency noise is less irritating than mid/high-frequency sounds. And for the material itself, a lower peak frequency can avoid exciting resonances. Lower-frequency vibrations are easier to damp with isolators and don’t cause rapid fatigue. The results of the analysis are shown in Figs 14 and 15.

From Fig 14, it can be seen that the effect of parameter b and parameter c on the response function is negative. And the Fig 15 represents the results of analyzing the effect between parameters for the response objective function.

Therefore, the relationship between the influence of each structural parameter on the performance of the metamaterial can be derived by the DOE method. Based on the influence factor of each parameter, researchers can more easily adjust the structural parameters to obtain the structure that meets specific sound insulation needs. However, for more complex structures or multiple structural parameters, there are limitations to this approach due to the interplay of performance between each parameter. The use of algorithms to optimize the performance of metamaterials, on the other hand, can be a good solution to the problem of multi-parameter tuning of complex structures.

### _Comparison of optimization effect based on genetic algorithm_

**Fig 16:**
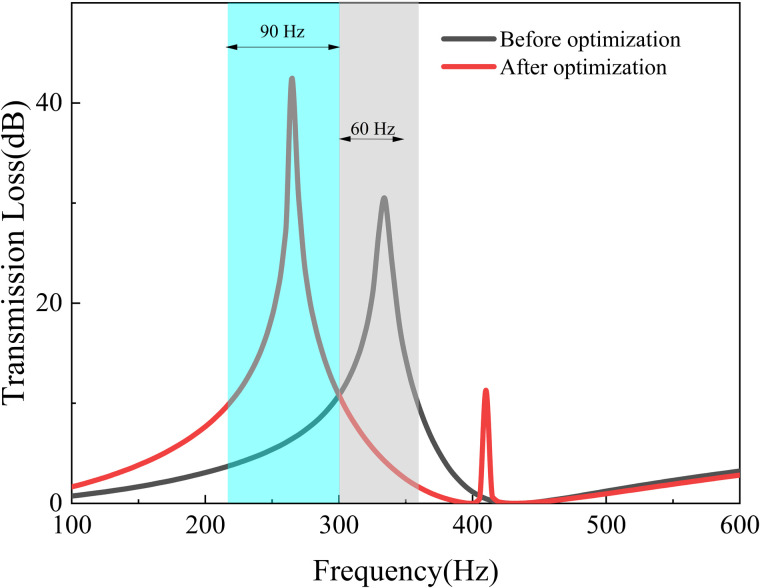
The comparison of TL before and after optimization.

In order to optimize the performance of TL, and can be set as optimization functions at the same time. In order to ensure the global optimization effect, this paper uses the multi-island genetic algorithm (MIGA) to automatically find the optimal calculation of the multi-structural parameters of metamaterials. The parameters of the optimization algorithm are shown in Table 4. The optimization process is shown in Fig 16.

**Table 4:** Parameters of muti-island genetic algorithm.

Parameters	Sub-population Size	Number of Islands	Number of Generations
	9	4	5
	Rate of Crossover	Rate of Mutation	Rate of Migration
	1	0.01	0.5

To optimize the performance of TL, 1.Maximizef(y) and 2.Maximizef(s) are set as optimization functions at the same time. In order to ensure the global optimization effect, the Multi-Island Genetic Algorithm (MIGA) is utilized in this paper for automatic optimization of metamaterial multi-structure parameters. The parameters of the optimization algorithm are shown in Table 4. The optimization result is shown in Fig 16.

where the total population = the product of the number of sub-totals and the number of islands. In general, the total number of populations usually ranges from 20 to 200.

Crossover rate: the higher the crossover probability, the higher the crossover rate of new individuals in the population. If the crossover rate is too low, the search may stagnate. Crossover rates typically range from 0.6 to 1.0.

Mutation rate: The mutation rate indicates the mutation status of an individual, and should not be too high, the default value is 0.01.

Migration rate: The rate at which populations are exchanged between islands is known as the migration rate, and is usually set between 0 and 0.5, and the default value is 0.5.

The comparison of the acoustic metamaterial TL before and after optimization is shown in Fig 16. It can be seen that the sound isolation bandwidth of the MIGA-optimized structure over 10 dB is in the range of 215–305 Hz with a bandwidth of 90 Hz. The optimized structure reaches the peak of 42.49 dB at about 265 Hz. And the original structure has a maximum peak of 40.78 dB at 335 Hz. Its bandwidth over 10 dB is 60 Hz, in the 300–360 Hz range. The optimization of the metamaterial structure shifts the peak frequency of the insulation by 70 Hz to lower frequencies and increases the bandwidth of the insulation by 30 Hz.

Thus, the sound insulation bandwidth is effectively increased and the optimization effect is 20.9%. The peak frequency is also shifted to lower frequencies to achieve superior low-frequency sound isolation, and the optimization effect of the algorithm reaches 50%.

NSGA-II is an improved non-dominated sorting genetic algorithm based on NSGA for multi-objective optimization [39,40]. Compared with the traditional genetic algorithm, NSGA-II expands the sampling space by introducing an elite strategy and has better global optimization capability. Moreover, it adopts computational metrics such as crowding degree and crowding comparison operator, which greatly improves the convergence speed of computational iterations.

The structural optimization of metamaterials usually involves multiple optimization objectives. Therefore, the multi-objective optimization algorithm has better applicability while taking into account the peak frequency, peak amplitude, and overall performance. The NSGA-II algorithm parameters are set as shown in Table 5:

**Table 5:** Parameters of NSGA-II algorithm.

Parameters	Population Size	Number of Generations	Crossover Probability
	36	5	0.9
	Crossover Distribution Index	Mutation Distribution Index	
	10	10	

In order to minimize the computational effort and accelerate the convergence speed, the population size is usually between 20 and 200. In this paper, the number of hereditary generations is set to 5. The crossover rate is usually between 0.6 and 1.0, which is set to 0.9 in this paper, and the crossover distribution index and mutation distribution index are the default values of the system.

**Fig 17:**
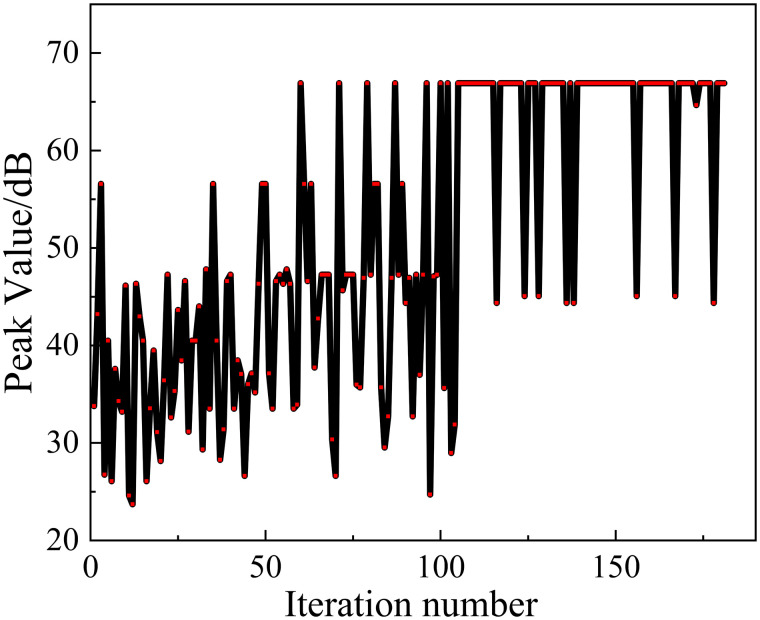
The process of the optimization (NSGA-Ⅱ).

**Fig 18:**
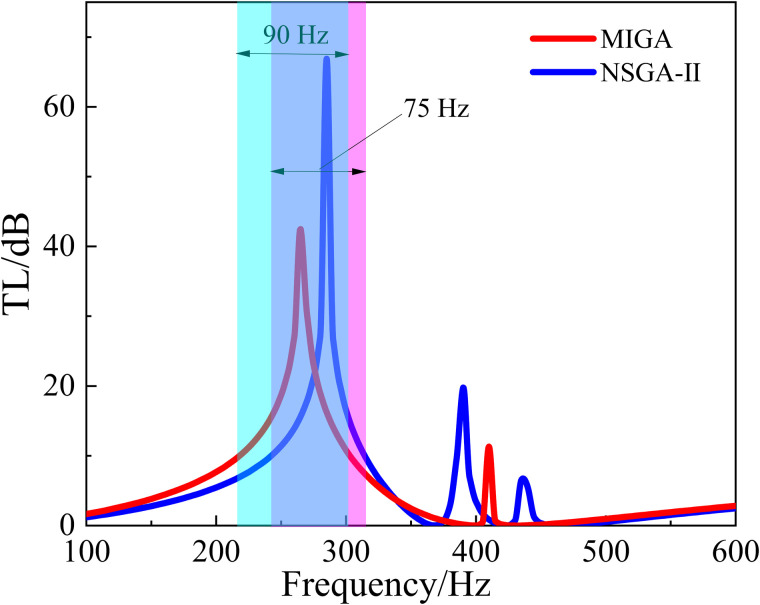
The comparison of different algorithm optimizations for TL.

The optimization functions are set as 1.Maximizef(y) and 2.Maximizef(s), and the optimization process and performance are shown in Figs 17 and 18. And the shaded area in Fig 18 indicates the region where the TL exceeds 10 dB.

It can be seen from Fig 17 that the algorithm converges at about the 100th cycle. From Fig 18, it’s shown that the sound isolation bandwidth of the optimized structure (NSGA-II) over 10 dB is in the range of 240–315 Hz with a bandwidth of 75 Hz. The structure reaches the peak of 66.9 dB at about 285 Hz. Compared with the original structure, the peak frequency of sound insulation has been shifted by 50 Hz to the lower frequency, and the sound insulation bandwidth has been increased by 15 Hz. Thus, the optimization effect of sound insulation bandwidth is 25%, and the optimization effect of peak frequency is 14.9%.

Compared with the original structure, both the MIGA and the NSGA-II algorithm have good optimization effects. Although the NSGA-II algorithm is slightly worse than the MIGA algorithm, the convergence of this algorithm is better, which improves the optimization efficiency.

**Fig 19:**
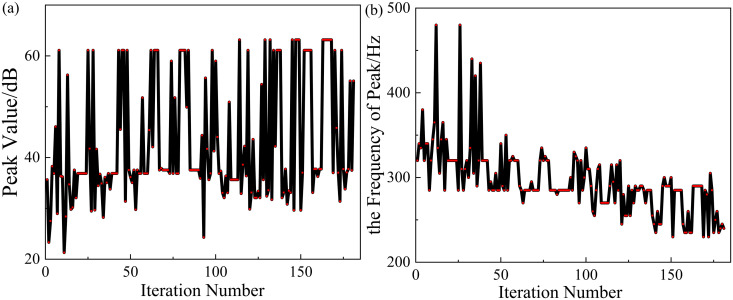
The process of the optimization (MIGA).

**Fig 20:**
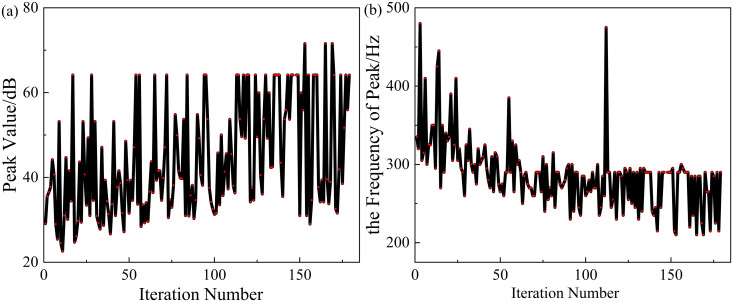
The process of the optimization (NSGA-Ⅱ).

**Fig 21:**
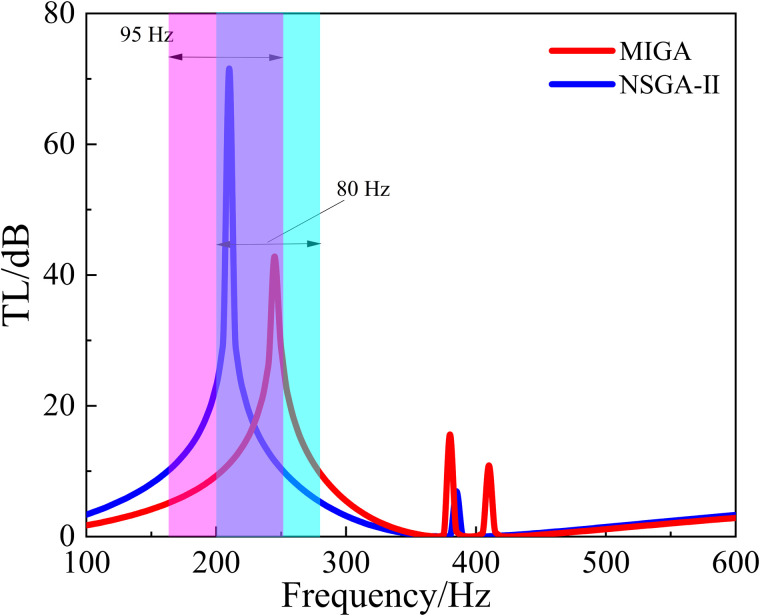
The comparison of different algorithm optimizations for TL.

In order to achieve better sound insulation effect of the metamaterial in the low-frequency range, 1.Maximizef(y) and 2.Minimizef(x) are set as the optimization objectives at the same time. And the optimization process of NSGA-II and MIGA is shown in Figs 19 and 20. The shaded area in Fig 21 indicates the region where the TL exceeds 10 dB.

It can be seen from Fig 21 that the optimized structure (MIGA) has a sound insulation of more than 10 dB in the frequency range of 200–280 Hz with the bandwidth of 80 Hz. The structures optimized for NSGA-II have a sound isolation of more than 10 dB in the 160–255 Hz frequency range with a bandwidth of 95 Hz. And the structure (NSGA-II) reaches a peak of 71.6 dB at approximately 210 Hz.

After the optimization of these two algorithms, the acoustic isolation peak of the metamaterial is shifted to the lower frequency, and the bandwidth of the acoustic isolation is also increased. After MIGA optimization, the frequency peak is shifted by 105 Hz towards the lower frequencies, and the bandwidth is increased by 20 Hz compared to the original structure. The optimization effect of sound insulation bandwidth is 33.4%, and the optimization effect of peak frequency is 31.4%.

For the NSGA-II optimization, the peak frequency is shifted to lower frequencies by 125 Hz and the bandwidth is increased by 35 Hz compared to the original structure. The optimization effect (NSGA-II) of sound insulation bandwidth is 58.4%, and the optimization effect of peak frequency is 37.4%.

After comparison, the NSGA-II algorithm is better for the optimization objectives 1.Maximizef(y) and 2.Minimizef(x).

However, it can be seen from Figs 19 and 20 that although the optimization effect of the two algorithms is obvious, both algorithms do not converge in the end. In order to solve the problem of poor convergence of the global optimization algorithm, this paper proposes a combination algorithm optimization strategy for the structure optimization problem of acoustic metamaterials.

### _Optimization method based on the combination algorithm strategy_

Although the global optimization algorithms can search for globally optimal solutions over a wide range, local optimization search is weak. Although the local optimization algorithm is easy to converge, its optimization quality is limited by the initial data because of its tendency to fall into local optimization. Therefore, solving the problem of initial data quality can effectively improve the optimization effect of the local optimization algorithm.

Therefore, this paper combines the two optimization algorithms, firstly using the global optimization algorithm to obtain a better quality global solution, and then using the local optimization algorithm to carry out the secondary local optimization calculation based on the global better solution.

The advantage of this combination optimization method is that while obtaining a globally better solution and ensuring the quality of the algorithm optimization, it can effectively improve the optimization effect and optimization efficiency of the algorithm.

In the previous section, the optimization of the NSGA-II algorithm for the objectives 1.Maximizef(y) and 2.Minimizef(x). is more effective. Therefore, the optimization method based on the combined algorithm strategy in this section takes NSGA-II as the global optimization algorithm and the NLPQL algorithm as the local optimization algorithm. Among them, the core algorithm of NLPQL is SQP (Sequential Quadratic Programming). Its main idea is to expand the objective function according to the second-order Taylor’s equation, and linearize the constraints. So as to transform it into a quadratic programming problem, and find the next design point through the quadratic solution. The NLPQL algorithm has better convergence compared to other algorithms and it can be combined with other algorithms for optimization.

**Fig 22:**
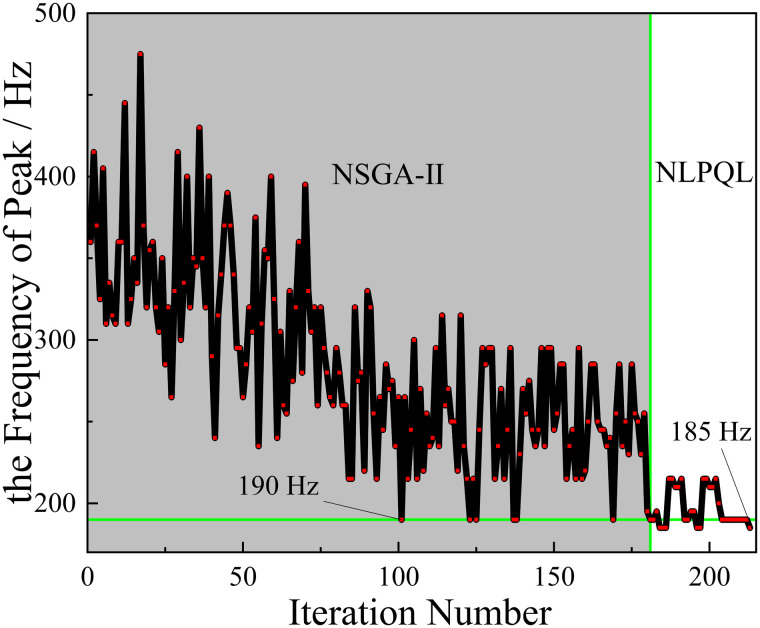
The process of the optimization (NSGA-Ⅱ+ NLPQL).

The optimization process based on the optimization strategy of the combinatorial algorithm is shown in Fig 22. Where the shaded part on the left is the optimization process of NSGA-Ⅱ and the right is the secondary optimization process of the local optimization algorithm. As can be seen from the Fig 22, in the combined optimization with NSGA-Ⅱ algorithm, the NLPQL algorithm converges and ends the optimization after 32 iterations. And by the optimization of NSGA-Ⅱ algorithm, the lowest peak frequency is 190 Hz. After the secondary optimization of NLPQL algorithm, the lowest peak frequency is reduced from 190 Hz to 185 Hz, which further improves the overall optimization effect of the algorithm.

**Fig 23:**
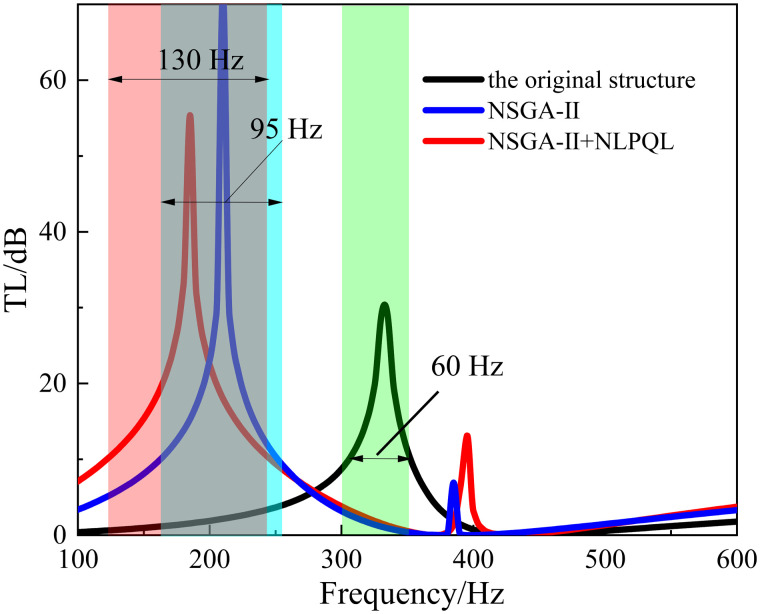
Comparison of optimization effects of combined algorithms (NSGA-Ⅱ+ NLPQL).

After optimization of the algorithm, its sound insulation performance is shown in Fig 23. The optimized structure (NSGA-II+NLPQL) provides more than 10 dB of sound isolation in the range of 120-250 Hz, with the bandwidth of 130 Hz. And it reaches the peak of 55.4 dB at about 185 Hz, while the original structure’s sound isolation band is located in the range of 300-360 Hz with the bandwidth of 60 dB; and it reaches a peak of 40.78 dB at 335 Hz.

Therefore, the bandwidth of the optimized structure (NSGA- II+NLPQL) has a bandwidth increase of 70 Hz and the optimization effect is nearly 117%. The peak frequency of acoustic isolation is reduced by 150 Hz, and the optimization effect is 44.8%. The optimization result shows that this algorithm combination optimization strategy can effectively regulate the resonance frequency of the metamaterial structure so that the peak frequency of sound insulation is shifted to the low frequency. The acoustic isolation bandwidth of the metamaterial is also greatly improved, which proves the effectiveness of the algorithm in regulating the properties of the metamaterial.

Compared to the optimization effect of the single algorithm (NSGA-II), the optimized structure (NSGA-II+NLPQL) increased the sound insulation bandwidth by 35 Hz, which improved the effect by 36.8%; the frequency of the sound insulation peak decreased by 25 Hz, which improved the optimization effect by 11.9%.

Therefore, by introducing the NLPQL algorithm combined with the genetic algorithm, not only can it solve the convergence problem of the global optimization algorithm well, but also plays the advantages of the global optimization algorithm to improve the optimization effect.

### _Optimization of additional mass block positions_

In order to further validate the effectiveness of the co-simulation method proposed in this paper, this section optimizes the position of the additional mass block on the membrane by means of the co-simulation method. The additional mass blocks are placed as shown in Fig 3b, which allows the resonance frequency of the metamaterial structure to be adjusted, and as such the low-frequency acoustic isolation performance of the metamaterial can be adjusted.

**Fig 24:**
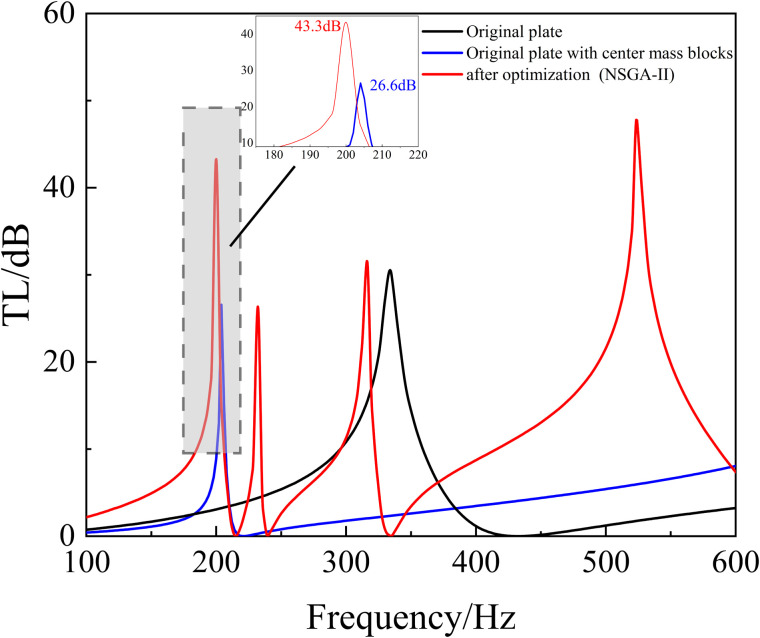
The comparison of TL before and after optimization.

The mass blocks are made of aluminum alloy with the radius of 2 mm and the height of 10 mm and their material properties are shown in Table 6. The calculated sound insulation performance of the metamaterial with the center mass block attached is shown in Fig 24 (blue line). After the addition of the mass block, the sound insulation peak of the structure is shifted from 340 Hz to the low frequency of 200 Hz, but the sound insulation interval of more than 10 dB is located in the range of 190–220 Hz, and the bandwidth is only 30 Hz. Therefore, although the sound insulation peak of the metamaterial with the attached center mass block is effective in isolating noises around 200 Hz, the bandwidth of the isolation is decreased.

**Table 6:** The material characteristics of the mass block.

Young’s modulus	Poisson’s ratio	Density
70Gpa	0.33	2700 kg/m^3^

Therefore, based on the metamaterial structure obtained from algorithm optimization (NSGA-II + NLPQL) in the previous section, the NSGA-II algorithm is used to optimize the position of the mass block, and the optimization results are shown in Fig 24.

It is obvious that after the optimization of NSGA-II (red line), the curve of TL has four acoustic isolation peaks in the low-frequency range, which are located at 200 Hz, 232 Hz, 316 Hz, and 524 Hz, and each of these peaks has a peak value of more than 20 dB.

In addition, the optimized metamaterial structure has sound insulation of more than 10 dB in the frequency ranges of 184–204 Hz, 228–232 Hz, 292–324 Hz, and 416–588 Hz, with a total bandwidth of 218 Hz. Its sound isolation bandwidth is increased by 158 Hz when compared with the original structure, and the optimization effect reaches nearly 363%. Compared with the optimization of the combined algorithm (NSGA-II + NLPQL) in the previous section, the bandwidth is increased by 88 Hz and the optimization effect is also very significant.

In this case, multiple sound isolation peaks appear. This is because changing the position of the membrane-attached mass block causes the metamaterial to produce additional resonance modes in the low-frequency range. Therefore, adjusting the position of the membrane-attached mass block based on the algorithm can better regulate the performance of the acoustic metamaterials to meet the specific acoustic isolation needs. And the optimization of the NSGA-II algorithm, the acoustic bandwidth of the metamaterial is greatly increased and the low-frequency performance is significantly improved. This also validates the effectiveness of the co-simulation method proposed in this paper,

Consequently, the application of the algorithm to optimize the structural parameters of metamaterials is effective. This also demonstrates the effectiveness of the DGN optimization method proposed in this paper based on co-simulation in regulating the acoustic properties of metamaterials.

## Conclusion

Although the global optimization algorithm has a strong global search ability within the design range of parameters, its local optimization ability is relatively weak. Although local optimization algorithms tend to converge easily, their optimization quality is limited by the initial data and they are prone to fall into local optimization.

Therefore, in this paper, for the sound insulation performance of metamaterials, an optimization method based on a combined algorithm strategy is proposed. The global optimization algorithm is combined with the local optimization algorithm. On the premise of ensuring the optimization quality by using the global optimization algorithm, the global superior solution is re-optimized through the excellent local optimization ability of the local algorithm, reducing the calculation time and improving the optimization effect of the algorithm. Among them, the global optimization algorithm is the genetic algorithm, and the local optimization algorithm selects the NLPQL algorithm. Compared with other algorithms, the NLPQL algorithm has better convergence and can be combined and optimized with other algorithms. After the combined optimization of NSGA-II and NLPQL algorithms, the low-frequency sound insulation performance of metamaterials has been further improved. The relationship between each design factor (structural parameter) and response function (performance index) of metamaterials can be obtained by using the DGN method. It solves the problem that the traditional algorithm makes it difficult to reflect the degree of influence of structural parameters on the optimization target in the optimization process. Moreover, this method combines the global optimization algorithm with the local optimization algorithm, which not only ensures the optimization quality but also improves the optimization effect of the algorithm and optimization efficiency.

The optimized metamaterial structure increases the sound insulation bandwidth by 70 Hz compared to the original structure, with an optimization effect of nearly 116.7%. The peak frequency of sound insulation is reduced by 150 Hz with an optimization effect of 44.8%. Compared with the optimization effect of the single algorithm (NSGA-II), the optimized structure increases the acoustic isolation bandwidth by 35 Hz, which improves the effect by 36.8%; the frequency of the acoustic isolation peak decreases by 25 Hz, which improves the optimization effect by 11.9%. The optimization effect is very significant, which verifies the effectiveness of the DGN method in metamaterial design and performance optimization. Furthermore, the global-local strategy proposed in this paper may also be applicable to datasets with a large computational volume and multiple local optimal solutions, such as natural language processing and computer vision.

In order to improve the computational efficiency, in this paper, the properties of double-layer plate metamaterials and through-hole thin film metamaterials are analyzed based on the single-cell structure. However, the single-cell structure cannot exhibit the coupling effect between large-area spread metamaterial structures. Therefore, further research and analysis on the acoustic performance of large-area spread metamaterial structures are needed. In addition, the simulation of metamaterials in this paper are mainly based on the impedance tube experimental device for research. Sound waves are vertically incident, but the noise in real life often comes from all directions. Therefore, further research is still needed on the sound insulation performance of this metamaterial structure for noise sources in different directions. Moreover, due to the limitations of the author’s field, this paper lacks an analysis of the parameter sensitivity in the algorithm, and thus its robustness cannot be proved. The deficiencies mentioned above await further study.

## Supporting information

S1 File Code and data. (RAR)
